# CD38-mediated Ca^2+^ signaling contributes to glucagon-induced hepatic gluconeogenesis

**DOI:** 10.1038/srep10741

**Published:** 2015-06-03

**Authors:** So-Young Rah, Uh-Hyun Kim

**Affiliations:** 1 Department of Biochemistry and National Creative Research Laboratory for Ca^2+^ signaling Network; 2 The Institute of Cardiovascular Research, Chonbuk National University Medical School, Jeonju, 561-182.

## Abstract

CD38 is a multifunctional enzyme for the synthesis of Ca^2+^ second messengers. Glucagon promotes hepatic glucose production through Ca^2+^ signaling in the fasting condition. In this study, we investigated the role of CD38 in the glucagon signaling of hepatocytes. Here, we show that glucagon induces cyclic ADP-ribose (cADPR) production and sustained Ca^2+^ increases via CD38 in hepatocytes. 8-Br-cADPR, an antagonistic cADPR analog, completely blocked glucagon-induced Ca^2+^ increases and phosphorylation of cAMP response element-binding protein (CREB). Moreover, glucagon-induced sustained Ca^2+^ signals and translocation of CREB-regulated transcription coactivator 2 to the nucleus were absent and glucagon-induced glucose production and expression of glucose-6-phosphatase (G6Pase) and phosphoenolpyruvate carboxykinase (Pck1) are remarkably reduced in hepatocytes from CD38^−/−^ mice. Furthermore, in the fasting condition, CD38^−/−^ mice have decreased blood glucose and hepatic expression of G6Pase and Pck1 compared to wild type mice. Our data suggest that CD38/cADPR-mediated Ca^2+^ signals play a key role in glucagon-induced gluconeogenesis in hepatocytes, and that the signal pathway has significant clinical implications in metabolic diseases, including type 2 diabetes.

CD38, a type II transmembrane protein, possesses ADP-ribosyl cyclase (ADPR-cyclase) and cyclic ADP-ribose hydrolase (cADPR-hydrolase) activities^1^,^2^. These two enzymatic activities are involved in the conversion of β-nicotinamide adenine dinucleotide (β-NAD^+^) first to cyclic ADP-ribose (cADPR) and then to ADP-ribose (ADPR)^3^,^4^,^5^. The metabolite cADPR is known to increase intracellular Ca^2+^ concentrations ([Ca^2+^]_i_) by releasing Ca^2+^ from intracellular stores or by Ca^2+^ influx through the plasma membrane Ca^2+^ channels in a variety of cells^6^,^7^,^8^,^9^. Additionally, CD38 produces another Ca^2+^ second messenger: nicotinic acid adenine dinucleotide phosphate (NAADP)^10^. The organelles targeted by NAADP for Ca^2+^ mobilization were identified as lysosome-like acidic granules^11^.

During starvation or hypoglycemia, glucagon induces glycogen breakdown and gluconeogenesis in the liver in order to release glucose into circulation^12^,^13^. Binding of glucagon to its receptor causes activation of a heterotrimeric, stimulatory G protein (G_S_), which subsequently leads to the interaction and stimulation of adenylate cyclase, elevation of cAMP levels, and activation of cAMP response element-binding protein (CREB)-regulated transcription coactivator 2 (CRTC2) and protein kinase A (PKA)^14^,^15^,^16^. A target of active PKA is CREB, which upon phosphorylation translocates into the nucleus and activates the transcription of gluconeogenic enzymes, such as glucose-6-phosphatase (G6Pase) and phosphoenolpyruvate carboxykinase (Pck1)^17^,^18^. Furthermore, glucagon increases cytosolic Ca^2+^ through the PKA-mediated phosphorylation of inositol-1,4,5-trisphosphate receptors (IP_3_R), leading to an increase in calcineurin activity and the consequent dephosphorylation of CRTC2, a CREB coactivator^19^. Recently, it has been reported that a calcium calmodulin-dependent kinase II (CaMKII) is activated in a calcium- and IP_3_R-dependent manner by cAMP and glucagon in primary hepatocytes and by glucagon and fasting *in vivo*^20^. Although these findings suggest that IP_3_-mediated Ca^2+^ signaling is important in glucagon-mediated gluconeogenesis, further details on the Ca^2+^ signaling in hepatocytes have not been specified.

In this study, we investigated whether CD38 is activated to produce a Ca^2+^ second messenger in the glucagon signaling of hepatocytes. Our data show that CD38 plays a critical role in glucagon-induced glucose production by producing cADPR in hepatocytes.

## Results

### Glucagon stimulates cADPR production through CD38 in hepatocytes

**Figure 1:**
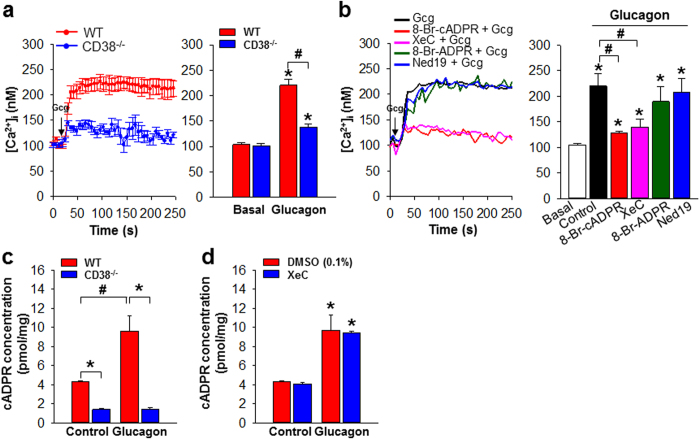
CD38 is involved in glucagon-induced cADPR production and [Ca^2+^]_i_ rise of HCs (**a**) HCs prepared from WT and CD38^−/−^ mice were loaded with fluo-4 AM and the changes in Ca^2+^ levels were measured using confocal microscope. The time point of 100 nM glucagon addition is indicated by the arrow. Data are mean ± SEM of [Ca^2+^]_i_ at 60 sec from five independent experiments. *P < 0.001 vs basal, ^#^*P* < 0.05. (**b**) Glucagon-induced [Ca^2+^]_i_ rise was prevented by 8-Br-cADPR (cADPR antagonist). 8-Br-cADPR (100 μM), 8-Br-ADPR (100 μM), Ned19 (100 μM), or XeC (2 μM) was preincubated for 20 min before the treatment with glucagon. The time point of 100 nM glucagon addition is indicated by the arrow. Data are mean ± SEM of [Ca^2+^]_i_ at 60 sec from five independent experiments. *P < 0.001 vs basal, ^#^*P* < 0.05. (**c**) HCs prepared from WT and CD38^−/−^ mice were treated with 100 nM glucagon for 15 sec, and then the concentrations of cADPR formed were measured with the cyclic enzymatic assay. Data are mean ± SEM of three independent experiments. *P < 0.001, ^#^*P* < 0.05. (**d**) HCs were treated with 100 nM glucagon for 15 sec after pretreatment with XeC (2 μM) for 20 min, and then the concentrations of cADPR formed were measured with the cyclic enzymatic assay. Data are mean ± SEM of five independent experiments. *P < 0.001.

It has been reported that glucagon increases intracellular Ca^2+^ concentration ([Ca^2+^]_i_), resulting in gluconeogenesis in hepatocytes (HCs). Firstly, we tested whether our prepared HCs are optimal condition by measuring [Ca^2+^]_i_ upon treatment with ATP, a well-known standard agonist^21^. The addition of ATP to the HCs from both wild type (WT) and CD38^−/−^ mice resulted in a rapid increase in [Ca^2+^]_i_, and the increased [Ca^2+^]_i_ was sustained (Supplementary Figure S1). To determine whether CD38 is involved in glucagon-induced Ca^2+^ signaling, we compared Ca^2+^ signals in glucagon-treated HCs from WT and CD38^−/−^ mice. Glucagon-mediated Ca^2+^ signals in WT HCs showed a rapid initial increase, which was followed by a sustained increase, and the latter was not observed in CD38^−/−^ HCs (Fig. 1a). This finding indicates that CD38 is involved in glucagon-induced Ca^2+^ signaling in HCs. Since CD38 produces many Ca^2+^ second messengers, such as cADPR, ADPR, and NAADP, and glucagon-induced Ca^2+^ signals show sustained Ca^2+^ increase, we further investigated which Ca^2+^ second messenger(s) were involved in glucagon-induced Ca^2+^ signals. Glucagon-induced Ca^2+^ increase was blocked by 8-Br-cADPR, an antagonistic analog of cADPR, but not by 8-Br-ADPR, an antagonistic ADPR analog, or Ned19, an NAADP mimic antagonist (Fig. 1b), indicating that cADPR is the Ca^2+^ second messenger responsible for glucagon-induced Ca^2+^ signals in HCs. Therefore, to examine whether cADPR is produced in HCs upon glucagon treatment, we compared cADPR levels in HCs from WT and CD38^−/−^ mice before and after treatment with glucagon. In response to glucagon treatment, cADPR levels were increased in HCs from WT mice, while cADPR was not produced in HCs from CD38^−/−^ mice (Fig. 1c). Moreover, basal levels of cADPR in HCs from CD38^−/−^ mice before treatment with glucagon was significantly lower than those in HCs from WT mice (Fig. 1c). These findings indicate that CD38 is responsible for the production of cADPR in glucagon-induced Ca^2+^ signals of HCs.

It has been previously shown that IP_3_-mediated Ca^2+^ signaling is involved in glucagon signaling of HCs^19^. To compare IP_3_- and cADPR-dependent glucagon-elicited Ca^2+^ signaling, we measured [Ca^2+^]_i_ in HCs treated with glucagon after pretreatment with Xestospongin C (XeC), an IP_3_ receptor antagonist. XeC inhibited glucagon-induced Ca^2+^ signaling (Fig. 1b). However, XeC did not affect glucagon-induced cADPR production (Fig. 1d). These findings indicate that glucagon receptor signaling activates PLC and CD38 with parallel, and their products, IP_3_ and cADPR, are prerequisite to glucagon-induced Ca^2+^ signals of HCs.

### cADPR-mediated Ca2^+^ signal regulates glucagon-induced glucose production and gluconeogenesis-related gene expression in HCs

**Figure 2:**
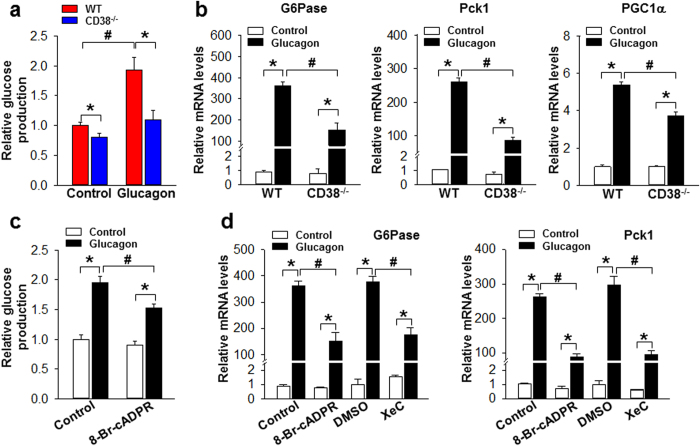
CD38 regulates glucagon-induced glucose production and gluconeogenesis related gene expression in primary HCs (**a**) HCs from WT and CD38^−/−^ mice were serum-starved overnight and then incubated with glucagon (100 nM) for 5 h in serum- and glucose-free media, and then glucose in the medium was assayed. Data are mean ± SEM of five independent experiments. *P < 0.01, ^#^*P* < 0.05. (**b**) Levels of mRNA were assayesd for G6Pase, Pck1, and PGC1α by real-time PCR after the treatment with glucagon (100 nM) for 5 h in HCs prepared from WT and CD38^−/−^ mice. Data are mean ± SEM of three independent experiments. *P < 0.01, ^#^*P* < 0.05. (**c**,**d**) 8-Br-cADPR inhibits glucagon-induced glucose production (**c**) and gluconeogenesis (**d**) related gene expression in primary HCs. HCs were serum-starved overnight and then incubated with glucagon for 5 h. 100 μM 8-Br-cADPR or 2 μM XeC was preincubated for 20 min before the treatment with 100 nM glucagon. DMSO was used with 0.1%. Data are mean ± SEM of three independent experiments. *P < 0.01, ^#^*P* < 0.05.

We next addressed the question of whether cADPR-mediated Ca^2+^ increase induces glucose production by measuring glucose levels in HCs from WT and CD38^−/−^ mice. The data showed that both basal and glucagon-induced hepatic glucose productions (HGP) were suppressed in CD38-deficient HCs (Fig. 2a). To further examine the role of CD38 in HGP, we investigated its transcriptional effects on genes encoding enzymes that regulate HGP, glucose-6-phosphatase (G6Pase), phosphoenolpyruvate carboxykinase (Pck1), and peroxisome proliferator-activated receptor gamma coactivator 1α (PGC1α). In all cases, glucagon-induced gene expression was lower in HCs from CD38^−/−^ mice than in HCs from WT mice (Fig. 2b). On the basis of the above observation that CD38 is definitely involved in HGP, we next examined the effects of cADPR on glucagon-induced HGP by treating HCs with 8-Br-cADPR, an antagonistic cADPR analog, and found that it indeed suppressed glucagon-induced HGP (Fig. 2c). Furthermore, pretreatment with 8-Br-cADPR significantly inhibited glucagon-induced mRNA expression levels of G6Pase and Pck1 (Fig. 2d). Consistent with the above observation of inhibition of glucagon-induced Ca^2+^ signaling by XeC treatment, XeC also inhibited glucagon-induced mRNA expression levels of G6Pase and Pck1 (Fig. 2d). These results indicate that cADPR produced by CD38 plays a critical role in glucagon-induced glucose production and gluconeogenesis-related gene expression in HCs.

### Glucagon stimulates CREB phosphorylation and CRTC2 translocation to nucleus in HCs in a CD38/cADPR-dependent manner

**Figure 3:**
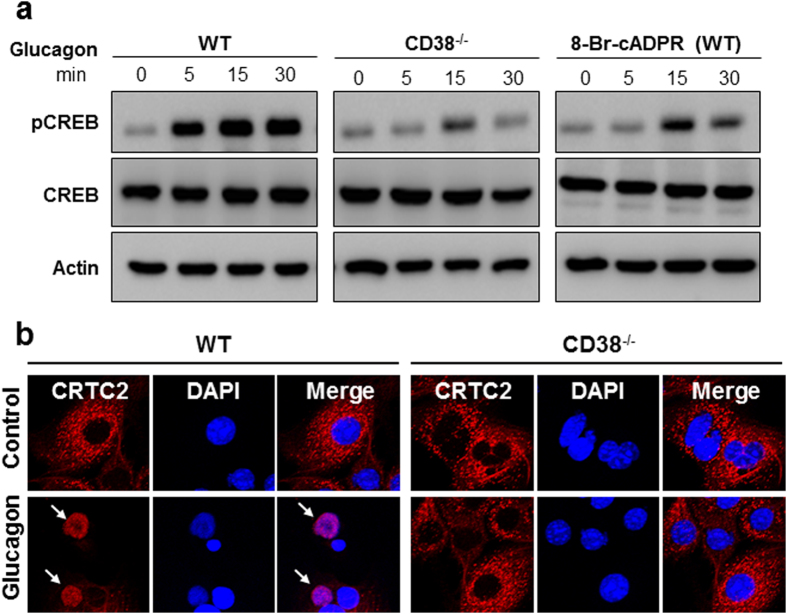
cADPR produced by CD38 activation is involved in glucagon-induced CREB phosphorylation and CRTC2 translocation to nucleus (**a**) CD38^−/−^ HCs and 8-Br-cADPR-treated HCs were prevented glucagon-induced phosphorylation of CREB. HCs prepared from WT and CD38^−/−^ mice were treated with 100 nM glucagon for indicated times, and lysis the cells, and then did westernblot about pCREB, CREB, and Actin. 100 μM 8-Br-cADPR, an antagonistic cADPR analog, was preincubated for 20 min before the treatment with 100 nM glucagon. (**b**) Glucagon stimulates CRTC2 translocation to nucleus via activation of CD38. HCs prepared from WT and CD38^−/−^ mice were treated with 100 nM glucagon for 30 min, and stained with anti-CRTC2 antibody (red), and then visualized with confocal microscope. Nucleus is stained with DAPI (blue). Shown is a representative of three independent experiments.

CRTC2 is regulated by changes in its localization between the cytoplasm and nucleus through Ca^2+^-dependent dephosphorylation in HGP^19^. Coincidentally, CREB phosphorylation is required for HGP, in concert with CRTC2^22^. We therefore explored the possibility that CD38/cADPR-mediated Ca^2+^ signals are involved in CREB phosphorylation and nuclear translocation of CRTC2 in glucagon signaling. CREB phosphorylation was induced as early as 5 min and sustained until 30 min, following treatment of WT HCs with glucagon, which was markedly reduced in CD38^−/−^ HCs (Fig. 3a). The glucagon-induced CREB phosphorylation was inhibited by pretreatment with 8-Br-cADPR (Fig. 3a). Moreover, glucagon-induced CRTC2 translocation to the nucleus was observed in WT HCs, while it was significantly reduced in CD38^−/−^ HCs (Fig. 3b). These results indicate that CD38/cADPR-mediated Ca^2+^ signaling regulates glucagon-induced CREB phosphorylation and the nuclear translocation of CRTC2.

### Decrease in blood glucose in association with decreased hepatic G6Pase and Pck1 mRNA levels in CD38^−/−^ mice

**Figure 4:**
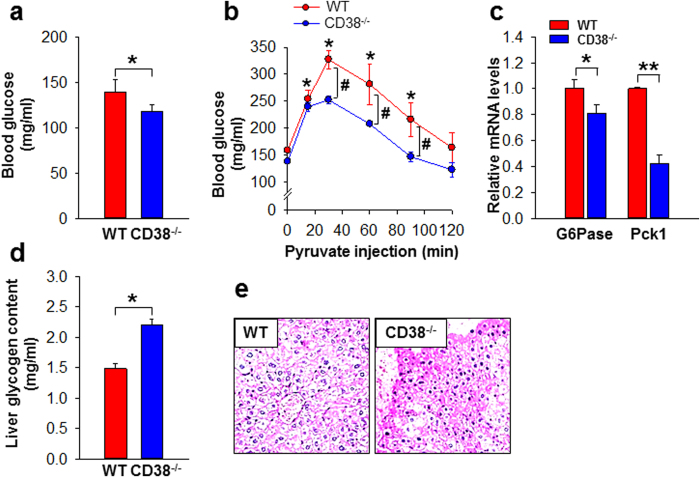
CD38 deficiency decreases blood glucose and hepatic G6Pase and Pck1 (**a**) Blood glucose of 18 h fasted 8-week-old WT and CD38^−/−^ mice (n = 5per cohort). *P < 0.005. (**b**) Pyruvate tolerance test. After mice were fasted for 18 h and challenged with pyruvate (2 g/kg body weight), blood glucose levels of mice were measured in indicated times (n = 5 per cohort). *P < 0.01 vs basal, ^#^*P* < 0.05. (**c**) mRNA levels of hepatic G6Pase and Pck1 were assayed with real-time PCR in 18 h fasted 8-week-old WT and CD38^−/−^ mice. Data are mean ± SEM of three independent experiments. *P < 0.01, **P < 0.001. (**d**) Liver glycogen content in fasted WT and CD38^−/−^ mice. Data are mean ± SEM of three independent experiments. *P < 0.001. (**e**) Periodic acid-Schiff (PAS) staining of liver sections in fasted WT and CD38^−/−^ mice. Shown is a representative of three independent experiments.

To assess the functional role of CD38 on hepatic glucose metabolism *in vivo*, we examined fasting blood glucose levels in WT and CD38^−/−^ mice. Consistent with our *in vitro* data, we observed a decrease in blood glucose levels in fasted CD38^−/−^ mice, compared to those in WT mice (Fig. 4a). The CD38^−/−^ mice also showed lower plasma glucose in response to a pyruvate challenge test (Fig. 4b). Consistent with data in primary HCs (Fig. 2b), there was a decrease in G6Pase and Pck1 mRNA levels in the livers of fasting CD38^−/−^ mice compared to those in WT mice (Fig. 4c). Because CD38^−/−^ mice showed a low response to glucagon in HGP, we compared the liver glycogen content in WT and CD38-deficient mice, and found the latter had significantly higher liver glycogen content than WT mice (Fig. 4d,e). These data further indicate that CD38 affects plasma glucose levels, pyruvate conversion into glucose, and the gene expression of gluconeogenic enzymes.

## Discussion

The current study investigated the role of CD38 in glucagon-induced Ca^2+^ signaling of HCs. Our data demonstrate for the first time that cADPR-mediated Ca^2+^ signals, via the activation of CD38, regulates hepatic gluconeogenic gene expression and HGP. Furthermore, primary HCs from CD38^−/−^ mice as well as treatment with 8-Br-cADPR leads to decreases in glucagon-induced CREB phosphorylation, translocation of CRTC2 to the nucleus, and hepatic gluconeogenic gene expression, resulting in reduced HGP.

CD38 is a multifunctional enzyme that produces many Ca^2+^ second messengers, such as cADPR, ADPR, and NAADP. Our current studies were focused on cytosolic Ca^2+^ signaling in hepatic gluconeogenesis, and our results showed that glucagon-mediated cytosolic Ca^2+^ increase is dependent on cADPR, but not on ADPR or NAADP (Fig. 1). Since CD38 is reportedly located in the nucleus of HCs^23^,^24^, it is quite interesting to see the role of nuclear CD38 in glucagon-mediated Ca^2+^ signaling as well as hepatic gluconeogenic gene expression.

Glucagon is an important hormone in the regulation of metabolism in the fasted state, particularly in promoting glucose release from HCs via gluconeogenesis and glycogenolysis. It has been reported that glucagon increases cytosolic Ca^2+^ through the PKA-mediated phosphorylation of IP_3_R^19^. CREB phosphorylation by PKA is prerequisite for enhancing the transcription of gluconeogenic enzymes^17^,^18^. Our finding clearly showed that CREB phosphorylation is dependent on CD38/cADPR-mediated Ca^2+^ signaling. Therefore, it is interesting to see how CD38/cADPR-mediated Ca^2+^ signaling regulates CREB phosphorylation in cooperation with cAMP/PKA signaling upon glucagon stimulation. Since IP_3_-mediated Ca^2+^ signaling is also coupled with cAMP/PKA signaling, it is important to elucidate details on the Ca^2+^ signaling, including the causal relationship between Ca^2+^ signaling messengers in glucagon-mediated gluconeogenesis of HCs.

Decreases in circulating insulin concentrations during fasting also stimulate gluconeogenic genes by the dephosphorylation of the forkhead activator FoxO1, which is one of the major regulators for fasting-induced hepatic gluconeogenesis^25^. Thus, we examined to see whether CD38-mediated signaling is also involved in this process and found that insulin-mediated FoxO1 phosphorylation was partially inhibited in CD38^−/−^ HCs compare with WT HCs (Supplementary Figure S2). In agreement with the less phosphorylation of FoxO1 in CD38^−/−^ HCs by insulin treatment, insulin could not block the gluconeogenic gene expression in CD38^−/−^ HCs (Supplementary Figure S2). These results indicate that CD38 plays a regulatory role in glucose metabolism at the multiple sites in the HCs.

Inhibition or genetic ablation of CD38 function has been correlated with reduced Ca^2+^ responses and insulin secretion in response to elevated glucose concentrations^26^, showing that CD38, which cyclizes NAD to produce cADPR, played a key role in insulin secretion. Further studies showed that another product of CD38, NAADP plays critical role in mediating the effects of the incretin, GLP-1, as well as high glucose^27^,^28^. Of note, islets from CD38-deficient mice shows not much impairment in insulin secretion upon stimulation with glucose or GLP-1 compared to wild type mice^28^, indicating that ADPR cyclases/NAADP-producing enzymes other than CD38 exists in β-cells. NAADP mediates Ca^2+^ signaling in insulin-stimulated glucose uptake in adipocytes and skeletal muscle^29^,^30^ and shows autocrine/paracrine function for glucose metabolism in β-cells and adipocytes^31^.The present study shows that CD38 plays a critical role in glucagon-induced glucose production by producing cADPR in hepatocytes. Collectively, these findings indicate that CD38 and its second messengers are important for glucose homeostasis.

It is also worth mentioning that our data specifying the roles of CD38/cADPR in glucagon-induced hepatic glucose production can have significant implications in the clinical management of hyperglycemic conditions, since a significant proportion of type 2 diabetic subjects have fasting blood sugar levels that are particularly difficult to control in spite of treatment that is otherwise adequate. These subjects can have disproportionally amplified hepatic glucose output, likely due to reduced sensitivity in the physiological suppression of glucagon production by hyperglycemia and/or GLP-1. A direct-acting therapeutic agent that reduces hepatic glucose production, combined with other effective antidiabetic regimens, would be an attractive clinical management option for these subjects.

In conclusion, we demonstrate that cADPR-mediated Ca^2+^ signals, via activation of CD38, plays a critical role in glucagon-mediated CREB phosphorylation, CRTC2 translocation to the nucleus, hepatic gluconeogenic gene expression, and glucose production in HCs. These data constitute a novel mechanism of Ca^2+^ signaling in glucagon-induced signaling, and may provide more options towards establishing therapeutic strategies against metabolic disorders.

## Methods

### Reagents

Antibodies were obtained as follows: CREB, phospho-CREB, FoxO1, and phospho-FoxO1 from Cell Signaling (Danvers, MA), CRTC2 from R&D Systems and Actin from Millipore. 8-Br-ADPR from Biolog Life Science Institute (Bremen, Germany) and Ned19 from Enzo Life Sciences (NY, USA), and all other reagents were obtained from Sigma-Aldrich (St. Louis, MO). Xestospongin C was from Santa cruz biotechnology.

### Mice

CD38 knockout mice (CD38^−/−^; B6.129P2-Cd38tm/Lud) were purchased from Jackson Laboratory (Bar Harbor, ME). Mice were bred and kept in animal housing facilities at Chonbuk National University Medical School under SPF conditions. All experimental animals were used under a protocol approved by the institutional animal care and user committee of the Chonbuk National University Medical School (CBU 2014-00031). Standard guidelines for laboratory animal care were performed in accordance with the Guide for the care and use of laboratory animals published by the National Institutes of Health^32^.

### Cell culture

Primary hepatocytes (HCs) were isolated from 8- to 12 weeks old male C57BL/6 J mice as described previously^33^. The inferior vena cava was cannulated and perfused at the rate of 4 ml/min. The portal vein was sectioned to allow flow through the liver. The liver was first perfused with Hanks’ balanced salt solution containing 0.5 mM EGTA and 10 mM Hepes at pH 7.4 followed by a perfusion with collagenase solution (0.5 mg/mouse Liberase (Roche) in Williams’ Medium E for 3 min at 4 ml/min. The liver was dissected, and HCs were isolated by mechanical dissection, filtered through a sterile 70-μm filter, and washed twice by centrifugation at 50 × *g* for 2 min. The HCs were cultured in Primaria plates (BD Biosciences, NJ, USA) with Williams’ Medium E (Life Technologies) containing 1% L-glutamine and penicillin/streptomycin and supplemented with 10% fetal bovine serum (Life Technologies). Four hours after plating, the medium was replaced with fresh medium. To experimental treatments, HCs were incubated overnight in serum-free Medium 199 (Life Technologies) before the addition of glucagon (100 nM).

### Glucose production in primary HCs

Glucose production assays were carried out as described^34^. After primary mouse HCs were harvested and cultured as described above, the cell culture medium switched to glucose- and phenol red-free DMEM (pH 7.4) supplemented the 20 mM sodium lactate and 2 mM sodium pyruvate. After 16 h of culture, 500 μl medium was collected, and glucose content was measured using a colorimetric glucose assay kit (BioAssay Systems, CA, USA). The readings were then normalized to the total protein amount in the whole-cell lysate.

### Glycogen content measurement

Glycogen content were measured carried out as described^35^. Livers (100 mg) were homogenized in 1 ml of H_2_O with protease and phosphatases inhibitors. Samples were then mixed KOH (1:2), boiled for 25 min, and washed with 70% ethanol. The pellet was dried and dissolved in 100 μl H_2_O, and the glycogen content was assessed using the glycogen assay kit (BioAssay Systems, CA, USA) according to the manufacturer’s instructions.

### PAS staining of mouse liver tissues

Liver tissues were fixed in 10% formalin for 24 h and embedded in paraffin. Sections (5 μm) were stained for glycogen using PAS stain (Sigma-Aldrich) according to the manufacturer’s instructions. The sections were counterstained with hematoxylin and examined by light microscopy.

### Pyruvate tolerance test

Mice were fasted overnight and injected intraperitoneally with pyruvate (2 g/kg body weight). Tail vein blood was taken for glucose measurement at 0, 15, 30, 60, 90 and 120 min after pyruvate injection. Blood glucose values were determined using a LifeScan automatic glucometer.

### Quantitative RT-PCR

Total cellular RNAs from whole liver or from primary HCs were extracted using the RNeasy kit (Qiagen, Valencia, CA). cDNA was synthesized by reverse transcription from 50 ng total RNA using a cDNA Reverse Transcriptase Kit (TaKaRa, Japan). The PCR reaction was carried out in 384-well plate using the ABI Prism 7900HT Sequence Detection System (Applied Biosystems). Real-time PCR primers for mouse G6Pase, Pck1, PGC1a and GAPDH were as follows: G6Pase (forward, 5′-CGACTCGCTATCTCCAAGTGA-3′, and reverse, 5′-GTTGAACCA GTCTCCGACCA-3′); Pck1 (forward, 5′-AAGCATTCAACGCCAGGTTC-3′, and reverse, 5′- GGGCGAGTCTGTCAGTTCAAT -3′); PGC1alpha (forward, 5’-TGTAGCGACCAATCGGAAAT-3’, and reverse, 5’-TGAGGACCGCTAGCAAGTTT-3’); GAPDH (forward, 5′-CGTCCCGTAGACAAAATGGT -3′, and reverse, 5′- TTGATGGCAACAATCTCCAC-3′).

### Measurement of [Ca^2+^]_i_

Changes of [Ca^2+^]_i_ in HCs were determined as described previously^19^. HCs on collagen-coated confocal dish were incubated with 5 μM Fluo-4 AM (Invitrogen) in media Medium 199 containing 1% BSA at 37 °C for 40 min after serum-starved for 24 h. The cells were washed three times with Medium 199. Changes of [Ca^2^^+^]_i_ were determined at 488 nm excitation/530 nm emission by air-cooled argon laser system. The emitted fluorescence at 530 nm was collected using a photomultiplier. The image was scanned using a confocal microscope (Nikon). For the calculation of [Ca^2+^]_i_, the method of Tsien *et al.*^36^ was used with the following equation: [Ca^2+^]_i_ = Kd(F − Fmin)/(Fmax − F), where Kd is 345 nM for fluo-4, and F is the observed fluorescence level. Each tracing was calibrated for the maximal (Fmax) by addition of ionomycin (8 μM) and for the minimal intensity (Fmin) by addition of EGTA 50 mM at the end of each measurement.

### Measurement of intracellular cADPR concentration

cADPR was measured by some modification of the cycling method described previously^37^. Briefly, cells were treated with 0.3 ml of 0.6 M perchloric acid under sonication. Precipitates were removed by centrifugation at 20,000 × g for 10 min. 3 volumes of perchloric acid extract were mixed with one volume of 2 M KHCO_3_, and vortexed. The resulting KClO_4_ precipitate was removed by centrifugation at 20,000 × g for 10 min. Supernatant was adjusted to pH 8.0. To remove all contaminating nucleotides, the samples were incubated with the following hydrolytic enzymes overnight at 37 °C: 0.44 unit/ml nucleotide pyrophosphatase, 12.5 units/ml alkaline phosphatase, 0.0625 units/ml NAD glycohydrolase, and 2.5 mM MgCl_2_ in 20 mM sodium phosphate buffer, pH 8.0. Enzymes were removed by filtration using a Centricon-3 filter (Amicon). To convert cADPR to β-NAD^+^, the samples (0.1 ml/tube) were incubated with 50 μl of cycling reagent containing 0.3 μg/ml Aplysia ADP-ribosyl cyclase, 30 mM nicotinamide, and 100 mM sodium phosphate, pH 8.0, at room temperature for 30 min. The samples were further incubated with the cycling reagent (0.1 ml) containing 2% ethanol, 100 μg/ml alcohol dehydrogenase, 20 μM resazurin, 10 μg/ml diaphorase, 10 μM riboflavin 5′-phosphate, 10 mM nicotinamide, 0.1 mg/ml BSA, and 100 mM sodium phosphate, pH 8.0, at room temperature for 2 h. An increase in the resorufin fluorescence was measured at an excitation of 544 nm and an emission of 590 nm using a SpectraMax gemini fluorescence plate reader (Molecular Devices Corp.). Various known concentrations of cADPR were also included in the cycling reaction to generate a standard curve.

### Statistical Analysis

Data represent means ± standard error of the mean (SEM) of at least three separate experiments. Statistical analysis was performed using Student’s t-test. A value of P < 0.05 was considered significant.

## Additional Information

**How to cite this article**: Rah, S.-Y. and Kim, U.-H. CD38-mediated Ca2+ signaling contributes to glucagon-induced hepatic gluconeogenesis. *Sci. Rep.* **5**, 10741; doi: 10.1038/srep10741 (2015).

## Supplementary Material

Supplementary Information
