# Supplementary material for: CD38-mediated Ca2+ signaling contributes to glucagon-induced hepatic gluconeogenesis
Source: Sci Rep. 2015 Jun 3;5:10741. doi: 10.1038/srep10741 (PMC4454144; doi:10.1038/srep10741)
Supplement: Supplementary Information [file srep10741-s1.pdf]

Supplementary information

CD38-mediated Ca^{2+} signaling contributes to glucagon-induced hepatic gluconeogenesis

So-Young Rah[1] and Uh-Hyun Kim[1, 2]

[1]Department of Biochemistry and National Creative Research Laboratory for Ca^{2+} signaling Network, and [2]the Institute of Cardiovascular Research, Chonbuk National University Medical School, Jeonju, 561-182

Supplementary Figure 1

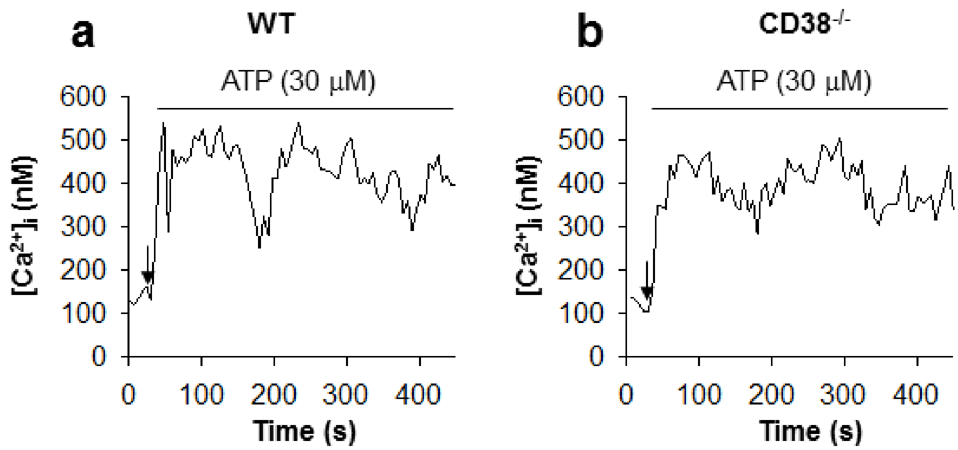

Figure S1. ATP-mediated Ca^{2+} signaling in WT and $CD38^{-/-}$ HCs. HCs prepared from WT and $CD38^{-/-}$ mice were loaded with fluo-4 AM and the changes in Ca^{2+} levels were measured using confocal microscope. The time point of 30 μM ATP addition is indicated by the arrow. n=10.

Supplementary Figure 2

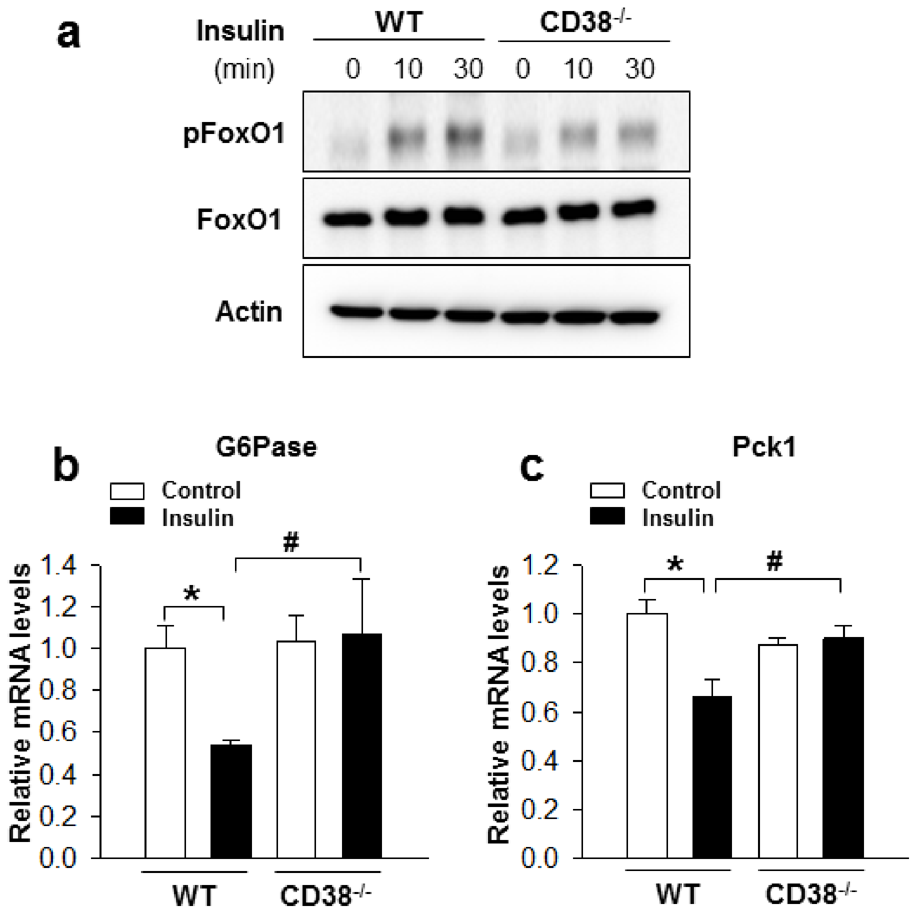

Figure S2. Insulin signaling in WT and CD38$^{-/-}$ HCs. (a) Phosphorylation of FoxO1 was assayesd after the treatment with insulin (100 nM) for indicated time in HCs prepared from WT and CD38$^{-/-}$ mice. (b, c) Levels of mRNA were assayesd for G6Pase and Pck1 by real-time PCR after the treatment with insulin (100 nM) for 3 h in HCs. Data are mean ± SEM of five independent experiments. *P < 0.01, $^{\#}P$ < 0.05.
